# Intraoperative Ocular Blood Flow Dynamics in Response to Intraocular Pressure Fluctuations During Vitrectomy for Proliferative Diabetic Retinopathy

**DOI:** 10.3390/jcm15052080

**Published:** 2026-03-09

**Authors:** Ryuya Hashimoto, Naoki Fujioka, Kazufumi Tanaka, Serika Moriyama, Takatoshi Maeno

**Affiliations:** Department of Ophthalmology, Toho University Sakura Medical Center, 564-1 Shimoshizu, Sakura 285-8741, Japan

**Keywords:** proliferative diabetic retinopathy, optic nerve head, autoregulation, blood flow, vitrectomy, laser speckle flowgraphy

## Abstract

**Background/Objectives:** This study aimed to evaluate the autoregulatory capacity of optic nerve head (ONH) tissue blood flow in response to intraocular pressure (IOP) fluctuations during vitrectomy in patients with proliferative diabetic retinopathy (PDR). We hypothesized that impaired autoregulation of ONH tissue blood flow in response to intraoperative IOP fluctuations could contribute to subsequent ONH atrophy and the development of visual field defects in PDR patients following vitrectomy. **Methods:** We included five eyes from five patients with PDR (mean age 70.6 ± 9.0 years) undergoing 25-gauge pars plana vitrectomy. ONH tissue blood flow was quantitatively assessed using intraoperative laser speckle flowgraphy. Mean blur rate in the tissue area (MT), an indicator of ONH tissue blood flow, was measured at baseline (infusion pressure 0 mmHg), during sustained elevation to 25 mmHg (at 5 and 10 min), and 1 min after return to baseline (11 min). IOP was modulated using the IOP Control system of the Constellation platform. **Results:** Elevation of IOP to 25 mmHg significantly reduced ONH tissue blood flow, with MT decreasing by 29% at 10 min compared with baseline (*p* < 0.05, Dunn’s multiple comparisons test). After IOP returned to baseline, MT significantly recovered compared with the 10 min measurement (*p* < 0.05) and returned to levels not significantly different from baseline (*p* > 0.05). **Conclusions:** MT decreases during intraoperative IOP elevation in PDR undergoing vitrectomy, but recovers after the return to baseline pressure, suggesting preserved short-term autoregulatory capacity. Careful IOP management during vitrectomy remains important in eyes with PDR.

## 1. Introduction

Diabetic retinopathy (DR) is the most common complication of diabetes mellitus and is responsible for approximately 3.7 million cases of visual impairment and more than 833,000 cases of blindness worldwide [1]. Proliferative DR (PDR) is known to lead to major complications, such as vitreous hemorrhage and retinal detachment due to the fibrovascular membrane (FVM) [2].

Pan-retinal laser photocoagulation (PRP) and anti-vascular endothelial growth factor (VEGF) therapy are effective therapies for PDR [1]. However, vitrectomy is required in cases of persistent vitreous hemorrhage or retinal detachment caused by fibrovascular membranes, despite prior PRP and anti-VEGF treatment. Post-vitrectomy complications in eyes with PDR include loss of visual acuity and visual field due to progressive optic atrophy, even after anatomically successful surgery, as reported in several studies [3,4,5]. Nevertheless, the mechanisms underlying optic atrophy following vitrectomy in PDR eyes remain incompletely understood.

One potential contributing factor is impaired autoregulation of ocular blood flow. Autoregulation is the ability of blood vessels to adjust blood flow in order to meet the metabolic demands of tissues [6]. In response to fluctuations in ocular perfusion pressure (OPP) caused by changes in systemic blood pressure or intraocular pressure (IOP), autoregulatory mechanisms function to maintain relatively constant blood flow [6]. In patients with DR, it is well established that this autoregulatory system is impaired due to endothelial cell dysfunction or loss of pericytes [7,8,9]. Feke et al. further reported that impaired autoregulation in response to increased OPP was associated with greater severity of DR [10].

Intraoperative fluctuations in IOP are a well-recognized phenomenon in ophthalmic surgery. During phacoemulsification, IOP may transiently exceed 60 mmHg, while in laser in situ keratomileusis, it can rise above 100 mmHg [11,12,13,14]. Although modern vitrectomy platforms, such as the Constellation^®^ Vision System, enable precise IOP control, typically maintaining a baseline of approximately 30 mmHg [15], surgery for PDR often requires deliberate, transient elevations in IOP. These elevations, frequently reaching 60 mmHg, are used to achieve hemostatic tamponade of bleeding neovascularization, with IOP promptly normalized following hemorrhage control [16]. Additional fluctuations may also occur due to surgical maneuvers, such as scleral depression during vitrectomy [17].

Such intraoperative IOP elevations during vitrectomy have been considered potential risk factors for ischemia of the optic nerve head (ONH) and retina [18,19]. We have previously reported that systemic conditions, such as diabetes mellitus or hyperglycemia, may impair ONH blood flow autoregulation in response to elevated IOP during vitrectomy, as assessed using a laser speckle flowgraphy–NAVI system (LSFG-NAVI) [20,21]. Given these findings and the documented occurrence of visual field defects following vitrectomy in eyes with PDR, evaluating intraoperative ONH blood flow in this patient population is of particular importance. While ONH tissue blood flow autoregulation in response to IOP elevation has been investigated in eyes with non-DR (NDR) and non-proliferative DR (NPDR), intraoperative ocular blood flow dynamics in eyes with PDR during vitrectomy have not been fully characterized.

Therefore, elucidating intraoperative blood flow dynamics in PDR may inform safer surgical strategies to prevent postoperative visual dysfunction and support the development of minimally invasive vitrectomy techniques that account for ocular circulation. The present study aimed to investigate changes in ONH tissue blood flow in response to IOP fluctuations during vitrectomy in eyes with PDR during vitrectomy using intraoperative LSFG-NAVI, which enables assessment of ocular blood flow in the supine position [20,21,22,23]. Additionally, we report changes in ONH tissue blood flow in a patient with PDR before and 5 months after vitrectomy.

## 2. Materials and Methods

### 2.1. Study Design and Participants

This retrospective clinical study was conducted at our hospital between December 2016 and March 2019. The research protocol adhered to the principles of the Declaration of Helsinki and was approved by the Ethics Committee at Toho University Sakura Medical Center (approval number: S24110_S24058, 5 March 2025). Information regarding the study was disclosed on the institutional website, providing potential participants with an opportunity to opt out.

The study included five eyes of five patients with PDR (mean age 70.6 ± 9.0 years; 2 men and 3 women). Patients were eligible for inclusion if they had PDR requiring vitrectomy, as defined by the Early Treatment Diabetic Retinopathy Study criteria [24]. Indications for surgery included non-clearing vitreous hemorrhage (*n* = 3) and significant fibrovascular membranes with or without tractional retinal detachment (*n* = 2) [25]. Patients were excluded if they had a history of ONH ischemic events, pre-existing optic neuropathies, concurrent glaucoma, previous vitreoretinal surgery, or refractive errors exceeding 6 D. Furthermore, patients were excluded intraoperatively if severe media opacity (e.g., dense vitreous hemorrhage) or extensive fibrovascular tissue over the optic disc precluded high-quality, unobstructed LSFG measurements.

### 2.2. Preoperative Assessment

Comprehensive ophthalmic examinations were performed, including assessment of best-corrected visual acuity, slit-lamp biomicroscopy, dilated fundus examination, and optical coherence tomography imaging. Systemic parameters, including blood pressure, fasting blood glucose, and glycated hemoglobin (HbA1c) levels, were recorded within 1 week preoperatively, consistent with previous studies evaluating ONH blood flow in patients with diabetes mellitus [20,21]. As part of our institutional protocol to minimize intraoperative bleeding, all five patients received an intravitreal anti-VEGF injection 1 day preoperatively.

### 2.3. Surgical Procedure and IOP Modulation

All surgeries were performed by a single experienced surgeon using the 25-gauge Constellation^®^ Vision System (Alcon Laboratories, Inc., Fort Worth, TX, USA). The irrigation solution consisted of BSS PLUS^®^ (Alcon, Fort Worth, TX, USA) supplemented with 0.5 mg epinephrine per 500 mL, a concentration previously shown to have no significant effect on ONH circulation [26]. Blood flow measurements were initiated approximately 30 min after administration of the retrobulbar block to minimize any acute effects of the anesthetic on IOP [27]. Following cataract surgery and core vitrectomy, a standardized IOP modulation protocol was implemented. IOP was controlled using the automated pressure control feature of the Constellation^®^ system (Alcon, Fort Worth, TX, USA), allowing continuous real-time monitoring. The initial infusion pressure was maintained at 0 mmHg to obtain stable baseline measurements. Subsequently, the pressure was elevated to 25 mmHg and maintained for 10 min, after which it was returned to the baseline pressure of 0 mmHg. Actual IOP was measured at each time point using a Tono-Pen AVIA^®^ (Reichert, Inc., Buffalo, NY, USA) to ensure accurate pressure readings. The IOP setting levels used in the present study (0 and 25 mmHg) fall within the physiological range commonly encountered during routine vitreoretinal surgery.

### 2.4. Blood Flow Measurements

In our department, intraoperative LSFG blood flow measurements are routinely performed during vitreoretinal surgery as part of standard patient care to monitor real-time ocular hemodynamics and minimize postoperative perfusion impairment, with particular attention to eyes at high risk of reduced perfusion, such as those with severe NPDR or PDR. ONH tissue blood flow was quantitatively assessed using intraoperative LSFG-NAVI (Softcare Co., Ltd., Fukuoka, Japan), which allows blood flow measurement in the supine position. LSFG is a noninvasive and quantitative method for assessing ONH blood flow based on analysis of changes in the speckle pattern of laser light reflected from the ocular fundus, expressed as mean blur rate (MBR) [28]. In addition, LSFG has been successfully used to evaluate blood flow in the ONH, retina, and choroid [29,30,31,32,33]. The LSFG data acquired intraoperatively were subsequently analyzed retrospectively using the LSFG Analyzer software (version 3.14.1.0; Softcare Co., Ltd., Fukuoka, Japan) to obtain quantitative blood-flow values.

In the present study, we analyzed the MBR in the tissue area (MT) to evaluate ONH tissue blood flow in eyes with PDR. This approach was based on previous findings showing that LSFG-derived MT reflects ONH tissue blood flow and that reduced MT may be associated with visual field progression in glaucomatous eyes [34]. Additionally, Aizawa et al. demonstrated that MT measured by LSFG was highly correlated with the capillary blood flow assessed using the hydrogen gas clearance method in both albino and pigmented rabbits. These findings suggest that tissue MBR might be suitable for interindividual and intergroup comparisons [35].

For each assessment point, three consecutive LSFG measurements were obtained and averaged to ensure reliability. The MT was recorded at four predefined time points intraoperatively: an initial baseline measurement at 0 mmHg after core vitrectomy; measurements at 5 and 10 min after IOP elevation to 25 mmHg; and a final measurement 1 min after returning to baseline pressure. Each LSFG acquisition was completed within 30 s to minimize surgical interruption. Throughout the procedure, systemic parameters, including blood pressure, heart rate, and oxygen saturation, were continuously monitored to ensure stable systemic conditions during the measurement period. All measurements were performed with patients maintained in the supine position.

### 2.5. Ocular Hemodynamics

Blood pressure and IOP were measured in all participants to calculate the OPP. Mean blood pressure (MBP) was calculated from systolic blood pressure (SBP) and diastolic blood pressure (DBP) using the following formula: MBP = 1/3 (SBP − DBP) + DBP. OPP in the supine position was calculated using the following formula: OPP (supine) = (115/130 × MBP) − IOP [36,37].

### 2.6. Systemic Parameters

IOP, blood pressure, pulse rate, and oxygen saturation (SpO_2_) were measured concurrently with ONH blood flow assessments. Blood pressure and pulse rate were measured using a bedside monitor (BSM-5132; Nihon-Kohden, Tokyo, Japan), and SpO_2_ was measured using a finger probe (TL-201T; Nihon-Kohden, Tokyo, Japan). All measurements were performed with patients in the supine position. Blood samples were collected within 1 month prior to the scheduled surgery. SBP and DBP were measured on the day before surgery and during surgery, and the timing of all LSFG-NAVI-OPE measurements was carefully recorded.

### 2.7. Statistical Analysis

Data are expressed as the mean ± standard deviation. Among the measured parameters, only the MT values were normalized by defining the baseline MT as 100%, with subsequent values expressed as percentages relative to baseline. Changes in normalized MT values were analyzed using a Friedman test followed by Dunn’s multiple comparisons test. All other parameters were analyzed using their original, non-normalized values. Statistical significance was defined as *p* < 0.05. All statistical analyses were performed using GraphPad Prism version 10.6.1 for macOS (GraphPad Software, San Diego, CA, USA).

## 3. Results

### 3.1. Patient Characteristics

The eyes of five patients with PDR (2 men and 3 women; mean age 70.6 ± 9.0 years) were included (Table 1). Three patients presented with persistent vitreous hemorrhage, and two presented with tractional retinal detachment as the primary surgical indication. The baseline clinical characteristics of the participants are summarized in Table 1. The mean body mass index was 23.4 ± 4.3 kg/m^2^, and the mean HbA1c level was 6.8 ± 1.1%. Three patients had concurrent hypertension, and two had hyperlipidemia.

### 3.2. Intraoperative Parameters

At baseline, the mean IOP was 8.60 ± 2.30 mmHg. Following elevation of the infusion pressure to 25 mmHg, the mean IOP increased significantly to 25.4 ± 5.37 mmHg at 10 min (*p* = 0.0423 for comparisons vs. baseline) (Table 2). After the infusion pressure was returned to 0 mmHg, mean IOP decreased to 8.20 ± 2.86 mmHg.

The mean blood pressure remained stable throughout the procedure (baseline: 97.2 ± 18.7 mmHg; 5 min: 96.8 ± 17.3 mmHg; 10 min: 93.9 ± 17.9 mmHg; return to baseline: 92.7 ± 13.9 mmHg; *p* = 0.083). The mean OPP significantly decreased from 77.4 ± 14.6 mmHg at baseline to 57.6 ± 17.2 mmHg at 10 min during IOP elevation (*p* = 0.0087 for comparisons vs. baseline). After returning to baseline pressure at 11 min, the OPP recovered to 73.8 ± 10.5 mmHg, with no significant difference from baseline values (*p* > 0.99). Throughout the procedure, both pulse rate (baseline: 78.8 ± 10.7 beats/min; final: 78.0 ± 11.6 beats/min; *p* = 0.447) and oxygen saturation (baseline: 94.0 ± 2.35%; final: 94.2 ± 2.05%; *p* = 0.906) remained stable, with no significant changes observed.

### 3.3. ONH Blood Flow Dynamics

**Figure 1:**
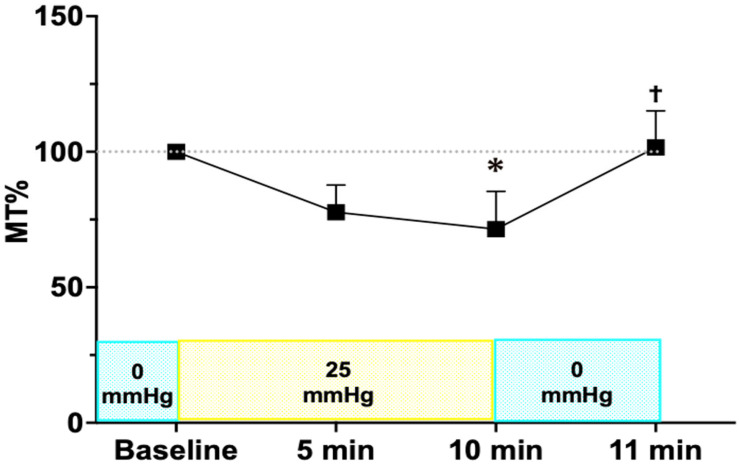
Changes in the mean blur rate in the tissue area (MT) in response to IOP elevation and restoration. MT decreased significantly at 10 min after IOP elevation to 25 mmHg (* *p* < 0.05 vs. baseline) and recovered to 102 ± 13.5% of baseline at 11 min after infusion pressure was returned to 0 mmHg († *p* < 0.05 vs. 10 min). Statistical analysis was performed using Dunn’s multiple comparisons test. Abbreviations: MT—mean blur rate in the tissue area.

The MT was 3.58 ± 0.98, 2.74 ± 0.58, 2.46 ± 0.30, and 3.56 ± 0.67 arbitrary units (AU) at baseline, 5 min, 10 min, and 11 min, respectively. When normalized to the baseline values, mean MT tended to decrease to 77.0 ± 10.0% at 5 min (*p* = 0.165) and further significantly declined to 71.5 ± 13.9% at 10 min during sustained IOP elevation (*p* = 0.042) (Figure 1). Individual reductions in blood flow ranged from 64.71% to 88.24% at 5 min and from 50.98% to 85.71% at 10 min. After returning to baseline IOP at 11 min, ONH tissue blood flow demonstrated significant recovery compared with 10 min measurement (*p* = 0.042), with mean MT reaching 102.0 ± 13.5% of baseline values (range, 87.2−121.0%).

### 3.4. Changes in ONH Tissue Blood Flow and Visual Outcome Following Vitrectomy in a Representative Case with PDR

**Figure 2:**
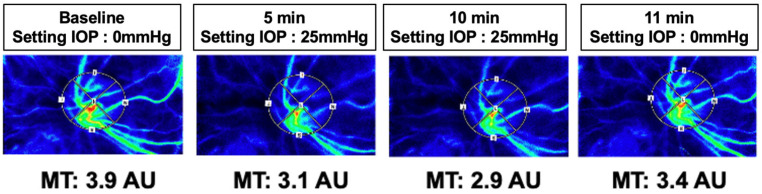
Representative LSFG composite color images showing changes in mean blur rate in the tissue area (MT) at baseline, during IOP elevation to 25 mmHg (5 and 10 min), and after IOP was restored to baseline (11 min). MT values decreased during IOP elevation and partially recovered after restoration of IOP. Abbreviations: LSFG—laser speckle flowgraphy; MT—mean blur rate in the tissue area; IOP—intraocular pressure.

Figure 2 presents representative LSFG images from a single case involving a 68-year-old Japanese woman with PDR who received an intravitreal anti-VEGF injection 1 day preoperatively. At baseline, with an actual IOP of 10 mmHg (infusion pressure set at 0 mmHg), the MT was 3.9 AU. Following elevation of the infusion pressure to 25 mmHg, the MT decreased to 3.1 AU (79.5% of baseline) at 5 min, when the measured IOP was 20 mmHg, and further declined to 2.9 AU (74.4% of baseline) at 10 min, with a measured IOP of 21 mmHg. After the infusion pressure was returned to 0 mmHg at 11 min, the MT recovered to 3.4 AU (87.2% of baseline) as the IOP returned to 10 mmHg.

**Figure 3:**
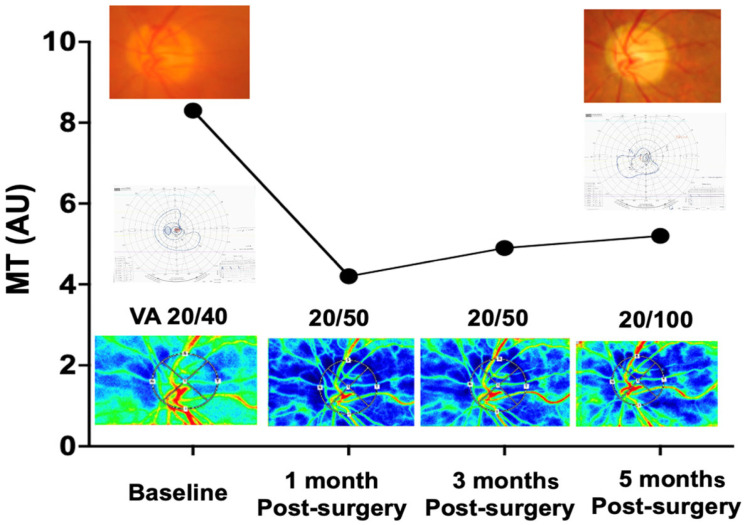
Time course of the mean blur rate in the tissue area (MT) before and after pars plana vitrectomy combined with panretinal photocoagulation in a patient with proliferative diabetic retinopathy exhibiting fibrovascular membrane. MT decreased postoperatively and showed gradual improvement over time, but did not fully return to baseline levels. Visual acuity declined progressively during the follow-up period, and optic disc pallor with atrophy was observed. Goldmann perimetry charts obtained preoperatively and at 5 months postoperatively are shown as ancillary clinical information, suggesting postoperative visual field change. Laser speckle flowgraphy images at baseline and during the postoperative period are shown below the graph. Abbreviations: MT—mean blur rate in the tissue area; VA−visual acuity.

Figure 3 illustrates the postoperative course of the same patient’s MT and clinical findings. The MT decreased from a preoperative baseline value of 8.3 AU to 4.2 AU at 1 month postoperatively. Although MT gradually improved to 4.9 AU at 3 months and 5.2 AU at 5 months postoperatively, these values remained significantly lower than the preoperative baseline, corresponding to 50.6%, 59.0%, and 62.7% of baseline, respectively. Despite the absence of recurrent fibrovascular proliferation or vitreous hemorrhage and an otherwise stable clinical course, the patient developed optic disc pallor and atrophy coinciding with the persistent reduction in MT. Visual acuity declined from 20/40 preoperatively to 20/100 at the final follow-up examination at 5 months postoperatively. Goldmann perimetry obtained preoperatively and at 5 months postoperatively suggested postoperative visual field change, including localized constriction in the superior temporal quadrant.

## 4. Discussion

The present study demonstrated a significant reduction in ONH tissue blood flow during controlled IOP elevation in patients with PDR undergoing vitrectomy. The MT significantly declined to 71.5 ± 13.9% at 10 min, highlighting the acute impact of elevated IOP on ONH perfusion. Upon the return of IOP to baseline, MT showed significant recovery compared with the 10 min measurement, reaching 102.0 ± 13.5% of baseline values.

Although the present study lacks a concurrent non-diabetic control group, our previous work using a similar IOP challenge in non-diabetic eyes demonstrated a significant recovery of ONH blood flow within 10 min of sustained pressure elevation [22]. The absence of such recovery during the period of elevated IOP in the present PDR cohort strongly suggests that autoregulatory function is more severely compromised in these patients.

Previous studies have highlighted retinal capillary pericytes in blood flow regulation, attributed to their contractile properties [8]. Early retinal changes in diabetes mellitus, including basement membrane thickening and pericyte loss, are well documented, and elevated glucose levels in retinal pericytes have been shown to induce resistance to normal regulatory mechanisms [8]. In addition, arteriosclerotic conditions, such as hypertension and hyperlipidemia, are known to impair autoregulation in patients with diabetes [7,21]. Our previous studies demonstrated that such arteriosclerotic conditions, including type 2 diabetes and hyperlipidemia, influence ONH blood flow autoregulation in eyes with NDR and mild NPDR [21]. Furthermore, impaired autoregulation has been shown to correlate with the severity of DR [10]. Taken together, and given that most patients with PDR have a history of hypertension or hyperlipidemia, it is plausible that ONH blood flow in PDR eyes was significantly reduced at 10 min during IOP elevation due to disrupted autoregulatory mechanisms.

In the present study, ONH tissue blood flow recovered substantially to approximately 102% of baseline values after IOP was returned to baseline. Autoregulatory capacity is known to be more impaired in patients with diabetes mellitus and poor control of hyperglycemia compared with those with well-controlled diabetes [8]. In addition, we previously demonstrated that higher HbA1c levels were associated with impaired autoregulation in ONH vessels of eyes with NDR and mild NPDR [21]. Moreover, in the present study, the mean HbA1c level was 6.8%, indicating well-controlled diabetes despite the advanced stage of DR. Considering these findings and the good glycemic control in our PDR group, the recovery of ONH tissue blood flow upon the restoration of IOP to baseline may be attributed to improved vascular compliance and the preserved capacity of the microvasculature to adapt to changes in IOP, even in the presence of temporarily impaired autoregulation.

In the representative PDR case, we observed a decrease in MT accompanied by the development of optic atrophy 5 months after vitrectomy combined with PRP. Previous studies have indicated that advanced stages of DR and renal dysfunction are associated with optic atrophy following vitrectomy [4]. Iwase et al. reported a significant correlation between the number of PRP sessions and reduced ONH vascular blood flow in patients with severe NPDR, suggesting that PRP itself may directly affect ONH blood flow [38]. In the present case, ONH tissue blood flow was reduced from 1 month after vitrectomy and PRP. The patient received an intravitreal anti-VEGF injection 1 day preoperatively, consistent with the treatment protocol used in the other cases in this study. VEGF has been shown to induce nitric oxide release [39], which plays a critical role in vascular regulation and blood flow control [40]. Conversely, anti-VEGF agents have been reported to reduce ocular blood flow [41,42]. Taken together, these findings suggest that disrupted ONH autoregulation during vitrectomy, potentially influenced by prior anti-VEGF therapy and PRP, may have contributed to persistent reductions in ONH tissue blood flow and the subsequent development of postoperative optic disc pallor.

The present study has some limitations. Firstly, the sample size was small. This small sample size precluded meaningful stratification by DR severity and multivariable adjustment for potential confounders. Further studies involving more patients with PDR are required to evaluate whether autoregulatory blood flow impairment worsens with advanced stages of DR in response to IOP fluctuations. Secondly, all patients received anti-VEGF treatment 1 day preoperatively and PRP during vitrectomy. Importantly, detailed quantitative information on prior PRP performed at outside institutions (e.g., number of laser spots, number of sessions, or total delivered energy) was not consistently available in our medical records. Therefore, we were unable to adjust for cumulative PRP exposure, and its potential impact on ONH perfusion should be evaluated in future prospective studies. In addition, the surgical complexity, including the removal of fibrovascular membranes in all cases, is another confounding factor that could influence ocular blood flow. The optic atrophy observed after vitrectomy may also be related to these treatments. Therefore, it remains unclear to what extent impaired autoregulatory blood flow in response to elevated IOP influences optic atrophy after vitrectomy. We primarily focused on MT because LSFG-derived MT has been reported to correlate with visual field defect severity [43]; however, MV was not analyzed in this report and may provide complementary information in future studies. Thirdly, most patients in the PDR group were receiving medications for hypertension, hyperlipidemia, or type 2 diabetes mellitus. One patient was treated with insulin, which affects ocular blood flow regulation [44]. These medications and insulin might influence autoregulatory blood flow in response to IOP fluctuations. Fourthly, intraoperative IOP was measured using a Tono-Pen AVIA^®^, which may overestimate IOP compared with Goldmann applanation tonometry [45], and might have slightly influenced the accuracy of OPP estimation. Finally, while we initiated blood flow measurements 30 min after the retrobulbar block to mitigate the acute effects on IOP, a time point by which the initial pressure spike is reported to have subsided [27], we cannot entirely exclude the possibility of minor, residual anesthetic effects on ocular hemodynamics.

## 5. Conclusions

ONH tissue blood flow autoregulation may be impaired in patients with PDR, potentially influencing postoperative outcomes. Our findings emphasize the importance of careful IOP management during vitrectomy in these patients to minimize the risk of further impairment, even though autoregulatory capacity appears to be preserved for short-term pressure fluctuations.

## Figures and Tables

**Table 1:** Clinical characteristics of the pilot study participant.

	PDR
Patients (n)	5
Men/women	2:3
Age (years)	70.6 ± 9.0
NDR/ mild NPDR	N/A
Vitreous hemorrhage	3/5
Body mass index (kg/m^2^)	23.4 ± 4.3
Systolic blood pressure (mmHg)	149 ± 30.1
Diastolic blood pressure (mmHg)	75.4 ± 16.6
Hypertension (%)	3/5
Hyperlipidemia (%)	2/5
Administration of Insulin (%)	1/5
Administration of ARB (%)	3/5
Administration of CCB (%)	1/5
Administration of diuretics (%)	0/5
Administration of statin (%)	2/5
Administration of antiplatelet drugs	1/5
Operating time (minutes)	126 ± 94.2
Injection of anti-VEGF drugs before surgery	5/5
Triglycerides (mg/dL)	126 ± 40.9
HDL cholesterol (mg/dL)	56.4 ± 15.3
LDL cholesterol (mg/dL)	99.8 ± 24.2
Fasting plasma glucose (mg/dL)	133 ± 25.8
Hemoglobin A1c (%)	6.8 ± 1.1

Abbreviations: NDR—non-diabetic retinopathy; NPDR—non-proliferative diabetic retinopathy; BMI—body mass index; ARB—angiotensin II receptor blocker; CCB—calcium channel blocker; VEGF—vascular endothelial growth factor; HDL—high-density lipoprotein; LDL—low-density lipoprotein; HbA1c—hemoglobin A1c.

**Table 2:** Changes in infusion pressure, mean intraocular pressure, mean blood pressure, ocular perfusion pressure, pulse rate, SpO_2_.

	N = 5			
	Baseline	5 min	10 min	11 min
Infusion pressure (mmHg)	0	25	25	0
Mean IOP (mmHg)	8.60 ± 2.30	24.6 ± 4.22	25.4 ± 5.37 *	8.20 ± 2.86 ^†^
mean MBP (mmHg)	97.2± 18.7	96.8± 17.3	93.9 ± 17.9	92.7 ± 13.9
Mean OPP (mmHg)	77.4 ± 14.6	61.0 ± 16.8	57.6 ± 17.2 *	73.8 ± 10.5 ^†^
Pulse rate (beats/min)	78.8 ± 10.7	77.8 ± 12.2	78.6 ± 11.2	78.0 ± 11.6
SpO_2_ (%)	94.0 ± 2.35	94.0 ± 1.87	94.0 ± 2.00	94.2 ± 2.05

IOP—intraocular pressure; MBP—mean blood pressure; OPP—ocular perfusion pressure; SpO_2_—oxygen saturation. * *p* < 0.05 vs. baseline. ^†^ *p* < 0.05 vs. at 10 min.

## Data Availability

The data presented in this study are available on request from the corresponding author due to privacy.

## Abbreviations

The following abbreviations are used in this manuscript:

ARB	Angiotensin II receptor blocker
AU	Arbitrary units
BMI	Body mass index
BSS	Balanced salt solution
CCB	Calcium channel blocker
DBP	Diastolic blood pressure
DR	Diabetic retinopathy
HbA1c	Hemoglobin A1c
HDL	High-density lipoprotein
IOP	Intraocular pressure
LDL	Low-density lipoprotein
LSFG	Laser speckle flowgraphy
LSFG NAVI	Laser speckle flowgraphy–NAVI system
MBP	Mean blood pressure
MBR	Mean blur rate
MT	MBR in the tissue area
NDR	Non-diabetic retinopathy
NPDR	Non-proliferative diabetic retinopathy
ONH	Optic nerve head
OPP	Ocular perfusion pressure
PDR	Proliferative diabetic retinopathy
PRP	Pan-retinal photocoagulation
SBP	Systolic blood pressure
SpO_2_	Oxygen saturation
VEGF	Vascular endothelial growth factor
